# Effects of controlled supramaximal high-intensity interval training on muscle capacities and physical functions for older adults: analysis of secondary outcomes from the Umeå HIT study—a randomised controlled trial

**DOI:** 10.1093/ageing/afae226

**Published:** 2024-10-13

**Authors:** Erik Frykholm, Mattias Hedlund, Clemens Becker, Henrik Holmberg, Bengt Johansson, Jochen Klenk, Nina Lindelöf, Ulrich Lindemann, Emma Simonsson, Carl-Johan Boraxbekk, Erik Rosendahl

**Affiliations:** Department of Community Medicine and Rehabilitation, Umeå University, 901 87 Umeå, Sweden; Department of Community Medicine and Rehabilitation, Umeå University, 901 87 Umeå, Sweden; Department of Geriatrics, Robert Bosch Hospital, Stuttgart, Germany; Unit Digitale Geriatrie, Medical Faculty of University Heidelberg, 69117 Heidelberg, Germany; Department of Epidemiology and Global Health, Umeå University, 901 87 Umeå, Sweden; Department of Surgical and Perioperative Sciences, Umeå University, 901 87 Umeå, Sweden; Department of Geriatrics, Robert Bosch Hospital, Stuttgart, Germany; Institute of Epidemiology and Medical Biometry, Ulm University, 890 75 Ulm, Germany; IB University of Health and Social Sciences, Study Centre Stuttgart, 700 49 Stuttgart, Germany; Department of Community Medicine and Rehabilitation, Umeå University, 901 87 Umeå, Sweden; Department of Geriatrics, Robert Bosch Hospital, Stuttgart, Germany; Department of Community Medicine and Rehabilitation, Umeå University, 901 87 Umeå, Sweden; Department of Diagnostics and Intervention, Diagnostic Radiology, Umeå University, 901 87 Umeå, Sweden; Institute for Clinical Medicine, Faculty of Medical and Health Sciences, University of Copenhagen, 2200 Copenhagen N, Denmark; Institute of Sports Medicine Copenhagen (ISMC) and Department of Neurology, Copenhagen University Hospital Bispebjerg, 2400 Copenhagen NV, Denmark; Department of Community Medicine and Rehabilitation, Umeå University, 901 87 Umeå, Sweden

**Keywords:** randomised controlled trial, high-intensity interval training (HIIT), sprint interval training (SIT), exercise, aged, older people

## Abstract

**Objective:**

This study investigated the effectiveness of supramaximal high-intensity interval training (supramaximal HIT) on muscle capacities and physical function compared to moderate-intensity training (MIT) for older adults.

**Methods:**

Sixty-eight older adults (66–79 years, 56% women), not engaged in regular exercise, were randomised to 3 months of twice-weekly supramaximal HIT (20 minutes including 10 × 6-second intervals) or MIT (40 minutes including 3 × 8-minute intervals). Both groups performed the training on stationary bicycles in a group setting. Target intensity was watt-controlled, with standardised cadence and individualised resistance. Outcomes analysed with linear-mixed models included leg power (Nottingham Power Rig), hand grip strength (Jamar dynamometer), static and dynamic balance (One leg stance, 30-second step test), chair stand (30-second chair stand), and anaerobic cycling performance (modified Borg Cycle Strength Test).

**Results:**

Baseline values were (supramaximal HIT/MIT, mean ± SD) leg power 198 ± 60/189 ± 53 W, hand grip strength 4.2 ± 1.0/4.3 ± 1.1 N/kg, static balance 64 ± 41/62 ± 41 s, dynamic balance 39 ± 7/38 ± 5 steps, chair stands 22 ± 6/22 ± 6 and anaerobic cycling performance 224 ± 60/217 ± 55 W. At 3-month follow-up, a between-group difference in favour of supramaximal HIT [95% CI] was observed in anaerobic cycling performance of 19[3;35] W. Within-group mean changes for supramaximal HIT/MIT were for leg power 8.4[0.9;15.8]/6.0[−1.3;13.3] W, hand grip strength 0.14[0.00;0.27]/0.13[−0.01;0.26] N/kg, static balance 11[3;20]/10[1;18] s, dynamic balance 1.6[0.3;2.8]/2.3[1.1;3.6] steps, 2.1[1.1;3.1]/1.4[0.4;2.3] chair stands and anaerobic cycling performance 31.3[19.6;43.0]/12.0[0.4;23.5] W.

**Conclusion:**

Supramaximal HIT showed superior effect on anaerobic cycling performance when compared to MIT. Additionally, the results indicate that supramaximal HIT is comparably beneficial as MIT in terms of effects on muscle capacity and physical function for older adults.

## Key Points

- Supramaximal high-intensity training (HIT) had similar effects as moderate-intensity training (MIT) on muscle function and functional performance.
- Supramaximal high-intensity training (HIT) had superior effects on anaerobic cycling performance compared to moderate-intensity training (MIT).
- The found effect on anaerobic performance may be especially relevant for older adults.

## Introduction

Older age often leads to a gradual decrease in physiological capacities such as cardiovascular function, muscle function and balance resulting in a general decline in physical capability. Regular physical activity and training are well-established factors that can prevent these impairments. However, despite compelling evidence, physical inactivity among older people remains a serious concern that needs to be addressed [1, 2].

High-intensity interval training (HIT) has been proposed as a time-effective training alternative with improvements in both physiological capacities and a variety of physical functions for middle-aged and older adults [3–6]. It is often performed in intervals ranging from 45 seconds to 4 minutes at high, but submaximal, intensity interspersed with recovery periods [7].

Interval training can also be programmed at supramaximal intensities (supramaximal HIT), which includes shorter work bouts at intensities greater than required to reach maximal oxygen consumption [7]. This form of exercise is less common but previous research indicate promising effects for older adults on muscle function, aerobic peak power output, and overall physical functioning [8–12]. However, the interpretation and implementation of these results are limited by small sample sizes, the use of inactive controls or lack of control conditions entirely, strict experimental settings and one-to-one supervision.

In the Umeå HIT Study, a randomised controlled trial, we observed a positive effect of group-based supramaximal HIT with watt-controlled target intensity on cardiorespiratory fitness and cardiovascular function, to a similar extent as moderate-intensity training (MIT), despite half the training time. Supramaximal HIT also revealed a superior improvement in muscle strength compared to MIT [13]. The additional finding regarding muscle strength is particularly noteworthy, given the known association between muscle function and physical function [14], and between physical function and future disability [15].

The aim of this study was to perform a preplanned secondary analysis of the effectiveness of group-based supramaximal HIT on muscle capacities and physical function, compared to MIT, for older adults not engaged in regular exercise.

## Methods

### Trial design

This study includes secondary outcomes of the Umeå HIT Study, a randomised controlled trial. Primary outcomes were cardiorespiratory fitness and global cognitive function; all secondary outcomes can be found at ClinicalTrials.gov (NCT03765385). The 30-second power output as an outcome of anaerobic cycling performance is an addition to the original study protocol. The Regional Ethical Review Board in Umeå, Sweden, approved the study (2018-307-31 M, 2018-421-32 M), and all participants provided their written informed consent before inclusion. Results are reported using the CONSORT 2010 checklist (Appendix 1).

### Participants

As previously described [13], volunteers were recruited for the study through newspaper advertising. The inclusion criteria were being 65 years old or older and not involved in regular exercise. The exclusion criteria were exercise-induced symptoms of movement-related dysfunction, symptomatic and unstable heart or lung conditions, untreated hypertension (≥140/90 mm Hg), insulin-treated diabetes, chronic and progressive neurological disease and a Mini-Mental State Examination score below 27. All participants underwent medical examinations and completed their baseline assessments including a peak oxygen consumption assessment before randomisation. The randomisation was stratified by age (65–69 years, or ≥ 70 years) and sex with a 1:1 group allocation, for each of the four training waves. An external researcher not involved in the study generated lists of random numbers using Research Randomiser (http://www.randomizer.org/).

### Intervention

See [13] for a detailed description of the intervention. In brief, the training for both groups included a warm-up, the supramaximal HIT or MIT intervals, and a cool-down. The controlled supramaximal HIT consisted of ten 6-second intervals interspersed with 54 seconds of recovery, totalling less than 10 minutes of actual interval training. Including warm-up and cool-down, each session lasted 20 minutes. The prescribed training intensity was based on the participant’s anaerobic cycling performance for 30 seconds with a modified Borg Cycle Strength Test described below. The maximum 6-second mean power output was estimated as 1.75 times the 30-second performance. The training intensity started at 60% of the maximum 6-second mean power output and could be individually progressed in absolute steps of four percentage points.

The MIT consisted of three 8-minute intervals interspersed with three minutes of recovery, totalling 30 minutes of moderate-intensity training. Including warm-up and cool-down, each session lasted 40 minutes. The prescribed training intensity for MIT was based on the participant’s maximal aerobic power during a standardised ramp test. Started at 40% of maximal aerobic power and could be individually progressed in absolute steps of four percentage points.

The training consisted of 25 sessions, conducted twice weekly, and was performed in groups at a local gym on stationary bicycles (Tomahawk IC7, Indoor Cycling Group, Nürnberg, Germany) for approximately three months. The first two sessions were introductory. The standardised criteria for progression of intensity allowed for individually adapted progression over time [13].

### Outcomes

All outcomes were assessed at baseline and 3-month follow-up at the Umeå University Sport Science Lab by an experienced physiotherapist. Outcomes were chosen to represent a range of physical functions that have been shown to be improved by physical activity and training, and important for physical capability [1].

### Muscle capacities

#### Leg power

Maximal leg extensor muscle power was assessed with the Nottingham Power Rig. Participants were seated with pelvic support against the backrest. They were instructed to push as fast as possible with a single leg against a moving plate over 16.5 cm. The instructor provided verbal encouragement at initiation of the movement. At the end of the movement, the leg was almost fully extended. Power was inferred from angular velocity and inertia of a flywheel. Out of at least five successive trials, the trial with the largest power of each leg was noted (W) [16]. The results of the right and left leg were averaged. For participants with complete values for only one leg at baseline or follow-up, the single value was used.

#### Hand grip strength

Maximal isometric hand grip strength was assessed using a Jamar hand dynamometer (Patterson Medical, Warrenville, IL). Participants were seated with the shoulder in an anatomical neutral position, elbow at 90 degrees, and wrist in a semiprone position. Participants were allowed a series of warm up trials, followed by two maximal trials for each hand. The participants were instructed to gradually increase force up to maximum force and hold this contraction for four to five seconds. The instructor provided verbal encouragement during the contraction. The participants were alternating between hands and there was a 1-minute rest period between trials. The best values in Newton for the left and right hand were averaged and then divided by body weight (N/kg). For participants with complete values only for one hand at baseline or follow-up, the single value was used.

### Physical function

#### Static and dynamic balance

Static balance was assessed with the one-leg stance test (barefoot and eyes open) [17]. The result was time, in seconds. For safety reasons were the participants placed in a corner facing the room. Participants were instructed to raise one foot approximately 10 cm from the floor without resting it on the supporting leg. Compensatory arm movement were permitted. They were informed about a maximum time limit of 120 seconds. Up to three trials were allowed per leg. The best results of the right and left leg in seconds were averaged.

Dynamic balance was assessed with a 30-second step test (with shoes). The test was initially developed to assess standing dynamic balance for stroke patients [18] and has been used for older adults with mild balance dysfunction [19]. The result was the number of times the participants could step on a 7.5-cm high stepping board and back to the floor in 30 seconds. The participants had to place the whole sole of the foot on the stepping board but needed only to touch the floor [18]. One trial per leg was used. The number of step-ups of the right and left leg were averaged.

#### Chair stand

The test leader demonstrated the Chair Stand test by performing a series of rapid chairstands and gave verbal instructions according to Jones, Rikli and Beam [20]. The test was conducted using a chair with a seat height of 43 cm, and the participants were instructed to cross their arms over their chest. One repetition was defined as extended legs at the top position and clearly touching the chair in the bottom position. Two trials were performed with at least 2 minutes of rest between. The number of stands completed within 30 seconds was noted and the best trial was used for analysis.

#### Anaerobic cycling

Mean power output for 30 seconds was assessed using a modified Borg Cycle Strength Test [21] on the same bikes as used in the training sessions. In our adapted version of the test, 30-second work stages were intercepted with 30 seconds of active rest. Workload increased by 45 watts and 36 W for each stage for men and women, respectively. A pedalling cadence of 85 revolutions per minute was maintained during each work stage. During the rest period, resistance was set to 0 and the pedalling cadence was held below 50 revolutions per minute. After each work stage, participants rated their perceived exertion using the Borg Rating of Perceived Exertion (RPE) scale [22]. The highest work stage where the participant could maintain the stipulated cadence with an RPE of ≤17 was noted as the 30-second power output.

### Blinding

Participants were informed about the study purpose but only received detailed information about their allocated training group. The same instructors managed both training groups. The outcome assessor was blinded to group allocation, and participants were repeatedly instructed to withhold any details of their group allocation during follow-up assessments.

### Sample size

Sample size estimation was based on estimated between-group differences in primary outcome variables peak oxygen consumption and global cognition with an estimated drop-out rate of 15%. A two-sided power calculation (80% power, α = 0.05) indicated a need for a total of 70 participants [13].

### Analyses

All analyses were conducted in R [23], and RStudio [24], using the tidyverse [25], lme4 [26], emmeans [27] and effectsize [28] packages. All available participant data from baseline and follow-up were included and analysed according to the original group allocation, regardless of adherence to the intervention, in agreement with the intention-to-treat principle. For each outcome, a linear mixed-effects model was applied to examine the between group difference in change, within-group change, and change over time irrespective of group. Group, time (baseline and 3-month follow-up), and a group × time interaction was used, with adjustment for age and sex as fixed effects, and individual as a random effect. Results were reported as linear mixed-effects model estimated mean change and difference in mean change, with 95% confidence interval (95% CI). A 95% CI excluding zero was chosen as indication of statistical significance. Effect size was calculated from the model estimate of the between-group difference in change divided by the unadjusted pooled SD.

## Results

**Figure 1:**
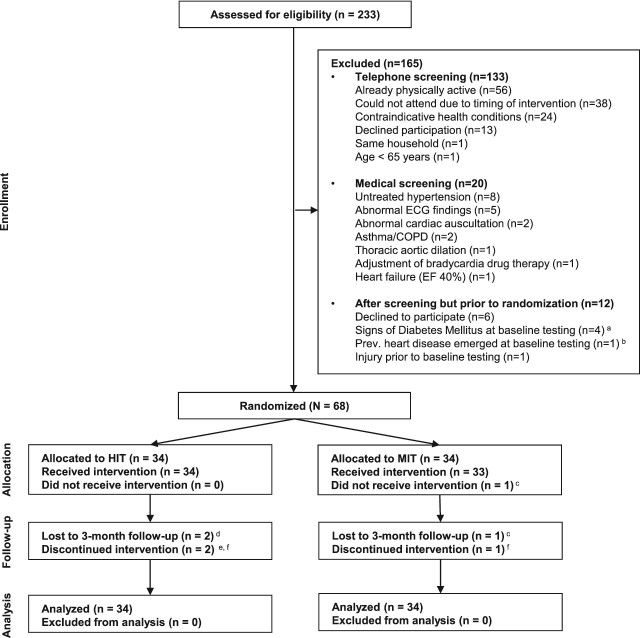
CONSORT flow diagram of Umeå HIT Study (from Simonsson et al. [13], copyright CC BY-NC). The enrolment in four separate waves started December 2018 and ended September 2019, the follow-up period started May 2019 and ended February 2020. COPD = chronic obstructive pulmonary disease; ECG = electrocardiogram; EF = ejection fraction; HIT = high-intensity interval training; MIT = moderate-intensity training. ^a^Signs of diabetes mellitus detected during baseline testing, necessitating further medical evaluation. ^b^Previously undisclosed heart disease identified during the baseline familiarisation visit. ^c^Participant withdrawal during the intervention phase before attending any exercise sessions. ^d^Participant withdrawal: one during the intervention and one after the intervention but before the 3-month follow-up. ^e^Exercise intervention discontinued upon physician's advice (due to re-diagnosis of thrombophlebitis) but participated in the 3-month follow-up. ^f^Exercise intervention discontinued at the participant's request but participated in the 3-month follow-up, in one case considered related to the intervention due to back pain after handling of equipment.

See Figure 1 for a study flow chart. Out of the 68 participants, 38 were women, 78% had some form of medical diagnosis, 56% were diagnosed with high blood pressure and 69% were on some type of medication. See Table 1 for baseline values of leg power, hand grip strength, static and dynamic balance, chair stands and anaerobic cycling performance.

**Table 1:** Participant characteristics at baseline

	All		Supramaximal HIT		MIT	
	*n*	Mean ± SD	*n*	Mean ± SD	*n*	Mean ± SD
Women, *n* (%)	68	38 (56)	34	19 (56)	34	19 (56)
Age, years	68	69.7 ± 3.0	34	69.7 ± 3.2	34	69.6 ± 2.8
BMI, kg/m^2^	68	26.2 ± 3.9	34	26.1 ± 3.5	34	26.4 ± 4.4
Vio2 peak, ml/kg/min	68	22.9 ± 4.9	34	23.6 ± 5.2	34	22.3 ± 4.6
Any diagnosis, *n* (%)	68	53 (78)	34	28 (82)	34	25 (74)
Hypertension, *n* (%)	68	38 (56)	34	17 (50)	34	21 (62)
Any medication, *n* (%)	68	47 (69)	34	23 (68)	34	24 (71)
Leg power, W	67	193.2 ± 56.2	33	197.5 ± 60.2	34	189.0 ± 52.7
Hand grip strength, N/kg	68	4.3 ± 1.0	34	4.2 ± 1.0	34	4.3 ± 1.1
Static balance, s	68	62.7 ± 40.5	34	63.6 ± 40.7	34	61.8 ± 40.9
Dynamic balance, *n*	68	38.3 ± 5.9	34	39.0 ± 6.5	34	37.6 ± 5.1
Chair stand, *n*	67	21.9 ± 5.6	33	21.8 ± 5.5	34	21.9 ± 5.7
Anaerobic cycling performance, W	68	221 ± 57	34	224 ± 60	34	217 ± 55

Note: BMI = body mass index; Vio2 peak = peak oxygen uptake; HIT = high-intensity interval training; MIT = moderate-intensity training; *n* = number of available measurements at baseline; SD = standard deviation

Exercise session attendance was 88.0% for supramaximal HIT and 88.1% for MIT. Out of a total of 1497 attended participant-sessions, participants reported experienced discomfort 52 times in the supramaximal HIT group and 94 in the MIT group. The majority were musculoskeletal. Importantly, none were considered a serious adverse event by external reviewers [13]. Additional information about adherence and experiences during training is presented in Frykholm et al. [29].

**Figure 2:**
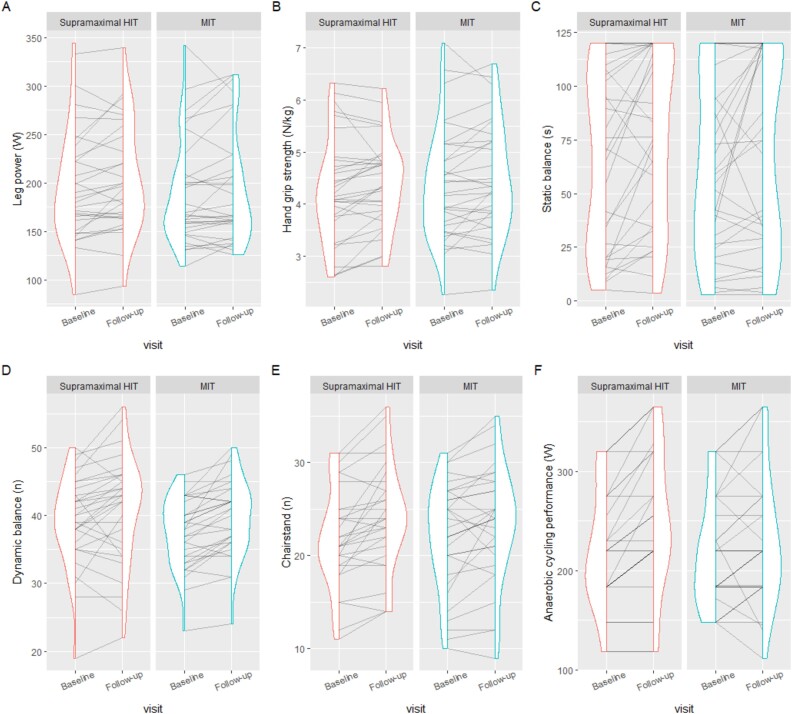
Vertical density plots that show a smoothed distribution of data at baseline and follow-up. Each line indicates the change of one individual from baseline to follow-up, (A) leg power (W), (B) hand grip strength (N/kg), (C) static balance (s), (D) dynamic balance (n), (E) chair stand (n), (F) anaerobic cycling (W). HIT = high-intensity interval training; MIT = moderate-intensity training.

At 3-month follow-up, a between-group difference in favour of supramaximal HIT [95% CI] was observed in anaerobic cycling performance of 19 [3; 35] W. No other significant between-group differences in change were observed. See Table 2 for within-group mean changes and between-group differences and Figure 2 for distributions of outcomes at baseline and follow-up and individual change.

**Table 2:** Within-group change and between-group differences in change from baseline to 3-month follow-up

				Within-group change						Between-group difference in change		
	All			Supramaximal HIT			MIT			Group × Time		
Outcome	n	Mean	[95% CI]	n	Mean	[95% CI]	n	Mean	[95% CI]	Mean	[95% CI]	ES
Leg power (W)	61	7.2	[2.0; 12.4]	30	8.4	[0.9; 15.8]	31	6.0	[−1.3; 13.3]	2.4	[−7.9; 12.5]	0.12
Hand grip strength (N/kg)	62	0.13	[0.04; 0.23]	31	0.14	[0.00; 0.27]	31	0.13	[−0.01; 0.26]	0.01	[−0.17; 0.2]	0.04
Static balance (s)	62	10.4	[4.2; 16.6]	31	11.2	[2.5; 20.0]	31	9.6	[0.9; 18.3]	1.6	[−10.4; 13.7]	0.07
Dynamic balance (n)	62	2.0	[1.1; 2.8]	31	1.6	[0.3; 2.8]	31	2.3	[1.1; 3.6]	−0.8	[−2.5; 1.0]	−0.22
Chair stand (n)	60	1.7	[1.0; 2.4]	29	2.1	[1.1; 3.1]	31	1.4	[0.4; 2.3]	0.8	[−0.6; 2.1]	0.28
Anaerobic cycling (W)	61	21.7	[13.4; 29.9]	30	31.3	[19.6; 43.0]	31	12.0	[0.4; 23.5]	19.3	[3.1; 35.3]	0.60

Note: Estimated marginal means from linear mixed-effects models adjusted for age and sex as fixed effects and individual as random effect. Reported as change within-groups, and between-group difference in change with 95% confidence intervals. Effect size was calculated from the model estimates of between-group difference in mean change and the unadjusted pooled standard deviation. *n* = number of available measurements at 3-month follow-up; CI = confidence interval; ES = effect size; HIT = high-intensity interval training; MIT = moderate-intensity training.

## Discussion

After three months of exercise, we observed a between-group difference in change in favour of supramaximal HIT over MIT in anaerobic cycling performance. Overall improvements were observed for all outcomes. The shown superior effects on anaerobic cycling performance by supramaximal HIT is important for older adults not engaged in regular exercise, as it may reflect an improvement relevant for daily activities.

Importantly, both groups improved in anaerobic cycling performance, with a greater improvement in favour of supramaximal HIT. This can likely be attributed to the overlap in demands of the training and assessment. Nevertheless, it suggests the potential of training at supramaximal intensities, which mirror the demands of some everyday activities. For instance, the relatively common task of climbing a flight of stairs can involve exertion at supramaximal intensity in this population [13, 30, 31]. By training at supramaximal intensities, participants can enhance their anaerobic capacity, thereby possibly improving their ability to perform such tasks. Previous participants of supramaximal HIT have in focus group interviews expressed increased self-confidence through this form of training. The self-confidence could be transferred to other forms of training as well as everyday life because they dared to, and were able to, exert themselves [32]. The mean improvement of 14% in anaerobic performance after supramaximal HIT in the current study is comparable with the results in a study with sedentary older men who performed six 30-second intervals at 50% peak power once every five days for six weeks. In that study, participants’ mean peak power output, measured by the Herbert 6-second peak power test [33], improved within-group by 17% [12].

The total mean improvement of around seven watts in leg peak power corresponds to about two years of anticipated decline for women and one year for men in this age group [34]. However, previous studies with older adults have shown larger improvements after equal or shorter training periods [35, 36]. Differences may be due to specificity of the training intervention [35], and larger training volume of high-intensity interval or moderate-intensity training [36]. Since the training interventions primarily targeted leg activity, the observed small mean improvement in hand grip strength is notable. It is nevertheless positive since higher levels of hand grip strength are indicative of better overall physical capacity and associated with a lower risk of mortality, regardless of age and follow-up period [37]. Previous studies of different types of interval training compared to MIT show somewhat similar results with small and comparable effects in intervention groups or no effects after training [6, 36, 38].

Improvements in both static and dynamic balance reached statistical significance in both groups. This can be an important training effect for older adults [39]. The clinical relevance may, however, be uncertain based on the modest improvements relative to baseline. The improvements may reflect better physical capability due to enhanced muscle function [14, 40]. Previous studies of HIT show both a lack of positive results in static balance [12, 41] and better results than in our study in dynamic balance [36]. This might be due to combinations of differences in assessment, training and population [12, 36, 41].

The nonsignificant difference between groups in chair stand in the current study agrees with the result from a recent systematic review and meta-analysis. It showed a small nonsignificant effect (ES = 0.20 95% CI [−0.17, 0.56] *P* = 0.28) in favour of any type of high-intensity interval training over moderate-intensity continuous training [5]. However, although not significantly different, the mean improvement in chair stand in the supramaximal HIT group in our study did reach a suggested minimal important change of two repetitions [42]. It should also be noted that the participants on average had a chair stand performance above reference values for older adults [43]; therefore, large improvements might be unlikely regardless of training intervention.

### Methodological discussion

Firstly, a selection bias is possible as participants interested in exercise and physical activity are more likely to report interest and be included in exercise interventions [44]. Nevertheless, we included participants not involved in regular exercise and without an upper age restriction. We did not exclude participants based on medical diagnosis but on risk. This resulted in a sample with participants up to 79 years of age with a lower mean peak oxygen consumption than in similar studies for this age group [8, 10–12] and 78% had at least one medical diagnosis.

Secondly, the sample size was initially calculated for primary outcomes [13]. Therefore, the power might be insufficient. Due to limited power to significantly establish small effects between training groups and the absence of a control group without training, results are also based on within-group comparisons. The choice of including an alternative training control group was partly based on ethical considerations regarding the known importance of physical activity and training for all older adults [1, 45]. The inclusion of participants in four waves ensured a limited seasonal effect of daily physical activity and strengthen the within-group analysis.

### Future research

The present study shows that the effectiveness of controlled supramaximal HIT for older adults is comparable to or greater than MIT. Comparisons with previous literature also indicate similarity in effectiveness with high-intensity interval training using longer intervals. The broad range of possibilities, however, adds complexity to decisions on what type of training to use when, for whom, and why. Future studies should aim to clarify the advantages and disadvantages of different types, including the preferences of older adults. Also, few existing protocols are designed with the clear ambition of broad implementation for older adults. This study is one of a few with older adults that perform supramaximal HIT with an approach to control the intensity to reduce exertion and discomfort [32]. This, in combination with performing the training in groups, increases opportunities for future implementation.

## Conclusion

Supramaximal HIT demonstrated superior effects on anaerobic cycling performance compared to MIT. The results also suggested that supramaximal HIT offers comparable benefits as MIT for improvements in various outcomes of muscle capacities and physical function. The shown superior effects on anaerobic cycling performance by supramaximal HIT is important for older adults, as it may reflect an enhancement relevant for daily activities.

## Supplementary Material

aa-24-0493-File004_afae226
